# Lumboperitoneal shunting as a rescue strategy in the management of complex cranial wound conditions

**DOI:** 10.1016/j.bas.2025.105869

**Published:** 2025-11-10

**Authors:** Christian Seemann, Jan Oros, Tobias Finger, Paul Kendlbacher, Sven König, Fatma Kilinç, Kristin Lucia, Christoph Hirche, Marcus Czabanka, Lina-Elisabeth Qasem, Vincent Prinz

**Affiliations:** a Department of Neurosurgery, Goethe University Frankfurt, University Hospital, Frankfurt am Main, Germany; b Department of Plastic, Hand and Reconstructive Microsurgery, BG Trauma Center Frankfurt am Main, Germany

**Keywords:** Lumboperitoneal shunt, Ventriculoperitoneal shunt, Wound healing condition, Complications, Complication management, Hydrocephalus

## Abstract

**Objective:**

Complex cranial wound conditions (CCWC), particularly when associated with hydrocephalus and/or implant-related infections, pose a major challenge in neurosurgical complication management. In such cases, moving the shunt system from the cranial to the lumbar compartment appears to be a valuable salvage strategy.

**Research question:**

Can lumboperitoneal shunting (LPS) including differential pressure and gravitational valves serve as an effective rescue strategy in patients with different types of CCWC?

**Methods:**

We conducted a single-center retrospective study of 15 patients treated with LPS implantation for CCWC between March 2023 and August 2025. Clinical data were extracted from medical records, including patient demographics, CCWC type, surgical parameters, complications and outcomes.

**Results:**

The cohort included 8 female and 7 male patients, with a median age of 53 years (range, 25–87). CCWC secondary to CSF fistulas following tumor resection accounted for 9 cases, VPS-associated infection for 3 cases, and decompressive craniectomy for 3 cases. In 5 patients, wound healing was further impaired by prior radiation and chemotherapy. Median surgical time was 60 min (IQR, 47–82), and median hospitalization was 6 days (IQR, 3–15). Wound healing resolved after LPS implantation in all but one patient. Complications occurred in 2 patients, both related to overdrainage. Implant survival rate was 100 %.

**Conclusion:**

LPS implantation represents a valuable salvage strategy for patients with CCWC, particularly in the context of decompressive craniectomy, radiation-exposed tissue, or prior implant-associated infection. LPS promotes cranial wound healing while ensuring CSF diversion, with adjustable gravitational valves being essential especially in craniectomized patients.

## Highlights

• Lumboperitoneal shunting resolves complex cranial wound conditions (CCWC).
• Salvage strategy avoids cranial hardware in high-risk patients.
• Adjustable gravitational valves minimize overdrainage complications.
• Implant survival rate was 100 % in 15 consecutive cases.
• LPS supports healing in radiation-damaged or infected cranial tissue.

## 1 Introduction

The management of complex cranial wound conditions (CCWC), particularly those associated with implant-related infections after neurosurgical procedures, remain a major clinical challenge and often necessitates multiple revision surgeries, including implant removal or exchange (Butenschoen et al., 2021; Ho et al., 2023; Aufschnaiter-Hiessboeck et al., 2024). A considerable proportion of these complications are linked to cerebrospinal fluid (CSF) circulation disorders, which may aggravate or perpetuate impaired wound healing (Sastry et al., 2022). When associated with hydrocephalus (Goedemans et al., 2020), these conditions are further compounded and often result in increased morbidity and mortality (Romualdo et al., 2025; Kurland et al., 2015). CSF fistulas, for example, occur in up to 30 % of patients following decompressive craniectomy (Goedemans et al., 2020; Romualdo et al., 2025; Kurland et al., 2015; Früh et al., 2023; Pfnür et al., 2024) or posterior fossa surgery (Sastry et al., 2022) and hydrocephalus develops in approximately 25–30 % of such cases (Goedemans et al., 2020; Romualdo et al., 2025; Kurland et al., 2015). Ventriculoperitoneal shunts (VPS) are widely used for CSF diversion but carry notable risks: implant-associated infections occur in 2–25 % of patients (Khalil et al., 2024; Conen et al., 2020; Renz et al., 2018; Olomo et al., 2023) and revision rates reach up to 24 % in tumor-associated hydrocephalus (Khalil et al., 2024; Olomo et al., 2023; Lu et al., 2023). Management of these complications frequently requires repeated or staged surgical interventions, temporary CSF diversion via lumbar or external ventricular drains, prolonged intravenous antibiotic therapy, and extended hospitalization – collectively imposing a substantial socioeconomic burden on patients and healthcare systems (Conen et al., 2020; Renz et al., 2018).

In cases where an infected VPS must be explanted, alternative CSF diversion strategies are necessary. Lumboperitoneal shunting (LPS) (Foo et al., 2023; Xie et al.; Yoon et al., 2017; Sinha et al., 2021; Li et al., 2022; Jusué-Torres et al., 2014; Li et al., 2024) offers a promising salvage option in selected patients, particularly in those with compromised soft tissue integrity or contraindications for cranial access. This includes patients with relevant frontal scaring after cranial surgery as well as a history of cranial radiation therapy and/or chemotherapy, which significantly increases the risk of wound complications due to soft tissue damage and impaired wound healing. Moreover patients with leptomeningeal carcinomatosis, who frequently require CSF diversion for hydrocephalus are at high risk for recurrent wound healing problems following VPS placement. In such scenarios, conventional revision strategies often fail to achieve stable wound healing (Foo et al., 2023; Zhang et al., 2015; Kim et al., 2025; Yamashiro et al., 2017). Here, we report our experience with LPS as a salvage approach in the treatment of CCWC. This strategy aims to reduce the incidence of recurrent infection, promote durable wound healing, and shorten hospitalization – ultimately alleviating both individual and systemic healthcare burden.

## 2 Methods and material

### 2.1 Study design

This single-center retrospective study was conducted at the Department of Neurosurgery, Goethe University Hospital, Frankfurt, Germany, and included all patients who underwent LPS implantation for the treatment of CCWC between March 2023 and August 2025.

### 2.2 Data collection

Clinical data were retrospectively extracted from electronic medical records in accordance with the General Data Protection Regulation (GDPR). Informed consent was waived under § 27 BDSG (Art. 9 GDPR, § 2(j)), which permits exceptions for scientific research. The study was approved by the institutional ethics committee and conducted in accordance with the Declaration of Helsinki. Written informed consent was obtained from all patients for the publication of photographic material.

Collected parameters included patient demographics, type of wound healing disorder (WHD), surgical revision strategies, duration of surgery, length of hospital stay, and complications.

### 2.3 Surgical procedure (LPS implantation)

A single-position lateral oblique approach, adapted from Goto et al. (2023) was used to optimize surgical workflow. Patients were placed on a vacuum mattress, which allowed for stable positioning and eliminated the need for intraoperative repositioning (Oros et al., 2025).

Three skin incisions were performed (Oros et al., 2025):

1. Midline paravertebral incision (for insertion of the spinal catheter)
2. Lateral supracostal incision (for valve board placement)
3. Paraumbilical incision (standard laparotomy for insertion of the peritoneal catheter)
1. **Spinal catheter placement:** A 2–3 cm paravertebral midline skin incision was made without opening the thoracolumbar fascia. A Tuohy needle was inserted at the L3/4 or L4/5 interspace. After confirming CSF flow, a spinal catheter was inserted cranially into the subdural space (15–20 cm from the subcutaneous entry) under fluoroscopic guidance to avoid kinking or caudal migration of the spinal catheter (Oros et al., 2025).
2. **Valve board placement:** A lateral supracostal subcutaneous pocket was prepared for placement of the valve board. The distal end of the spinal catheter was tunneled in an arcuate fashion to the subcostal pocket, where it was connected to the valve board and secured with a ligature. The valve board was anchored to the subcutaneous tissue using silk sutures. To allow better postoperative transcutaneous valve adjustments, the board was positioned superficially (Oros et al., 2025).
3. **Peritoneal catheter insertion:** A standard paraumbilical mini-laparotomy was performed to insert the distal peritoneal catheter using conventional surgical technique (Oros et al., 2025).

### 2.4 Preoperative considerations

LPS was only performed in patients with communicating hydrocephalus, as the presence of an obstructive component represents a contraindication for this procedure. In accordance with current neurosurgical standards, all patients underwent preoperative neuroimaging and clinical evaluation to rule out obstruction before LPS implantation. To ensure safety and treatment success, all patients were preoperatively evaluated according to clearly defined indications and contraindications.

Indications for LPS in the CCWC setting included:

– Presence of a communicating hydrocephalus
– Sterile CSF according to a standardized diagnostic protocol
– Feasible lumbar access, verified by preoperative CT or MRI of the spine to exclude anatomic barriers
– Uncompromised abdominal cavity, no history of intra-abdominal infection, adhesions, or other surgical contraindications to peritoneal catheter placement.

Contraindications comprised:

– Obstructive hydrocephalus
– Active infection involving the cranial wound, shunt system, or CSF according to a standardized diagnostic protocol
– Spinal deformities, prior instrumentation that prevents safe lumbar puncture or catheter placement
– Abdominal contraindications, such as peritonitis, abscess, or extensive intra-abdominal adhesions (bridles) that would compromise distal catheter positioning.

In patients with a history of VPS-associated infection or exposed cranial shunt components suggestive of underlying infection, LPS implantation was only considered after confirmed eradication of infection.

A **standardized diagnostic protocol** was applied to evaluate CSF parameters prior to LPS placement:

CSF cell count below 10 cells/μL, protein concentration below 2.0 g/L, lactate level below 2.1 mmol/L, at least two negative microbiological cultures and negative CSF PCR.

If all parameters were within normal range, a single-stage LPS implantation was performed. In cases of abnormal findings, LPS implantation was postponed and scheduled as a second-stage procedure.

We followed a **standardized therapeutic protocol** for both strategies:

Single-stage LPS implantation: VPS explantation and LPS implantation were performed during the same procedure, followed by a two-week course of oral antibiotics (cotrimoxazole or clindamycin).
Two-stage LPS implantation: Initial VPS explantation was followed by pathogen-directed intravenous antibiotic therapy for two weeks. Upon normalization of CSF parameters per protocol, LPS implantation was carried out as a second surgical step, followed by an additional two-week course of oral antibiotics.

Implant-associated infections were treated according to a standardized antibiotic protocol (Conen et al., 2020). In cases of soft tissue deficiency, augmentation was performed according to protocol in collaboration with plastic surgery, using local flaps, tissue expansion, or free flaps.

### 2.5 Statistical analysis

Data were analyzed using GraphPad Prism (version 9.5.1; GraphPad Software, San Diego, CA, USA). Descriptive statistics were calculated for all variables, with continuous data presented as means with standard deviations (SD) or medians with ranges, as appropriate. The normality of data distribution was assessed using the Kolmogorov-Smirnov test. Comparisons were conducted using the Mann-Whitney *U* test for nonparametric continuous data and the unpaired *t*-test for parametric continuous data. A p-value <0.05 was considered statistically significant.

## 3 Results

### 3.1 Patient demographics

**Fig. 1:**
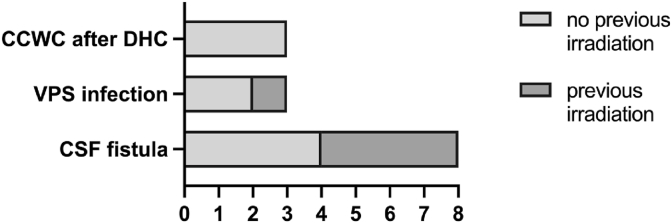
Type of CCWC and indication for LPS. **Abbreviations:** CCWC, complex cranial wound conditions; CSF, cerebrospinal fluid; DHC, decompressive hemicraniectomy; LPS, lumboperitoneal shunt; VPS, ventriculoperitoneal shunt.

The cohort included **8** female and **7** male patients, with a median age of 53 years (range, 25–87). CCWC secondary to CSF fistulas following tumor resection was found in 9 cases and in 3 patients CCWC was preceded by VPS-associated infection. Three patients developed severe CCWC after decompressive craniectomy. Among these patients, wound healing was further impaired in 5 patients due to prior radiation-induced skin damage. The median follow up time was 17 weeks (IQR, 4–28). Table 1 summarizes patient demographics and surgical course. Fig. 1 illustrates the type of CCWC and the indications for LPS implantation.

**Table 1:** Patient demographics and surgical course.

No.	sex	age	type of CCWC	type of HC	primary vs. secondary LPS implantation	primary disease	VPS prior to LPS	complications	number of surgeries to treat CCWC prior to LPS	surgical strategy	CCWC after LPS treated
1	f	25	CSF fistula + irradiated skin and chemo	malresorptive	primary	diffuse astrozytoma, WHO CNS grade 3	no	no	3	flap reconstruction	yes
2	m	33	CSF fistula (+CAD)	malresorptive	primary	meningioma, WHO CNS grade 2	no	no	5	dura + cranioplasty	yes
3	m	71	CSF fistula	malresorptive	primary	meningioma, WHO CNS 1	no	no	0	dura + cranioplasty	yes
4	f	35	CCWC after multiple recurrence surgeries + irradiated skin and chemo	leptomeningeal carcinomatosis	primary	glioma, NOS, CNS WHO grade 4	no	no	4	dura + cranioplasty	yes
5	m	53	CSF fistula	malresorptive	primary	olfactory groove meningioma, WHO CNS grade 1	no	no	0	duraplasty	yes
6	m	33	CSF fistula	malresorptive	primary	radiation-associated skull base osteomyelitis after nasopharyngeal carcinoma	EVD	no	3	endonasal duraplasty + skull base reconstruction	yes
7	m	37	CSF fistula + irradiated skin (+CAD)	malresorptive	primary	occipital brain metastasis	no	no	4	dura + cranioplasty	no
8	m		CSF fistula	malresorptive	primary	glioblastoma	no	no	1	duraplasty	yes
9	f	30	CCWC after DHC and posterior fossa decompression	posthemorrhagic	primary	TBI (+CVS)	EVD	no	0	cranioplasty	yes
10	m	53	CFS fistula + irradiated skin (+CAD)	malresorptive	secondary	osteomeningioma, WHO CNS grade 2	yes	overdrainage, peritoneal catheter displacement	8	flap reconstruction + split skin graft	yes
11	f	64	VPS infection + irradiated skin and chemo	leptomeningeal carcinomatosis	secondary	breast cancer	yes	no	5	flap reconstruction	yes
12	f	87	VPS infection	idiopathic	secondary	iNPH	yes	no	4	superficial wound revisions	yes
13	f	81	VPS infection	idiopathic	secondary	iNPH	yes	no	3	split skin graft	yes
14	f	49	CCWC after DHC (+ multiple CAD)	posthemorrhagic	secondary	SAH	yes	overdrainage	12	cranioplasty	yes
15	f	34	CCWC after DHC (+ multiple CAD)	posthemorrhagic	secondary	TBI (+osteonecrosis)	yes	no	3	cranioplasty	yes

**Abbreviations:** CAD, computer-aided designed cranioplasty; CCWC, complex cranial wound condition; CNS, central nervous system; CSF, cerebrospinal fluid; CVS, cerebral vasospasm; EVD, external ventricle drainage; HC, hydrocephalus; LPS, lumboperitoneal shunt; iNPH, idiopathic normal pressure hydrocephalus; TBI, traumatic brain injury; VPS, ventriculoperitoneal shunt; WHO, world health organization.

### 3.2 Surgical parameters and outcomes

The median surgical time was 60 min (IQR, 47–82). The median duration of hospitalization following LPS implantation was 6 days (IQR, 3–15) reflecting in most cases an uncomplicated postoperative course. Overall, the surgical outcome was favorable, with CCWC successfully resolved in all but one patient after LPS implantation. In that exceptional case, persistent cranial superficial wound problems required additional plastic reconstruction with a skin flap, which ultimately led to complete wound closure and stable healing with the LPS in situ.

Postoperative complications were observed in 2 of 15 patients (13 %). Both patients developed overdrainage, including one case complicated by subdural hygromas. Importantly, the hygromas were managed conservatively by valve adjustment, without the need for surgical intervention, and the patient made a satisfactory recovery. The second patient additionally experienced peritoneal catheter malpositioning, which necessitated revision surgery. In all cases, the LPS systems remained functional without failure throughout the follow-up period, providing durable CSF diversion and long-term wound stability. The overall implant survival rate was 100 %.

### 3.3 Representative Case 1: CSF fistula after meningioma resection – secondary LPS implantation

**Fig. 2:**
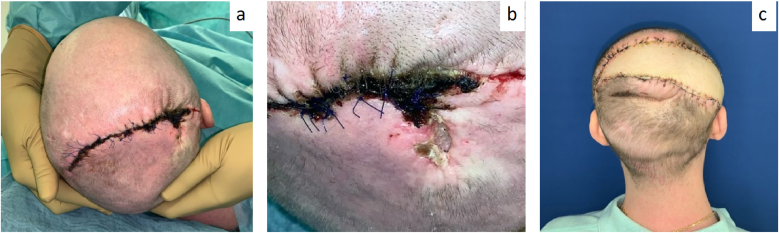
Representative Case 1: CSF fistula after meningioma resection – secondary LPS implantation. **2a.** Preoperative photo of a patient following tumor resection, complicated by CCWC and CSF fistula. **2b.** Preoperative photo showing wound dehiscence. **2c.** Postoperative result after multiple superficial wound revisions and local flap reconstruction.

A 33-year-old male patient with a large parietooccipital convexity meningioma, WHO CNS grade 2, underwent tumor resection. Due to massive intraoperative hemorrhage and brain swelling, the bone flap was not reinserted, and the patient subsequently developed a CSF fistula. After reimplantation of the bone flap, the fistula persisted and was complicated by wound infection, necessitating bone flap removal and free flap reconstruction. Following intravenous antibiotic therapy, an LPS was implanted, resulting in complete resolution of the CSF fistula. The patient later received a CAD implant during the clinical course and recovered well (see Fig. 2).

### 3.4 Representative Case 2: CSF fistula after meningioma resection – primary LPS implantation

**Fig. 3:**
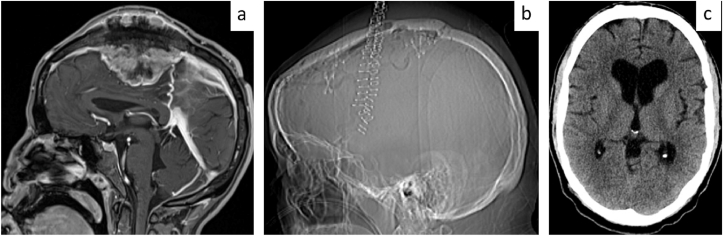
Representative Case 2: CSF fistula after meningioma resection – primary LPS implantation. **3a.** Contrast-enhanced T1-weighted cranial MRI demonstrating a large exophytically growing osteomeningioma. **3b.** Cranial CT scout image obtained after tumor resection and primary closure, including duraplasty and cranioplasty with palacos and microplate fixation. **3c.** Axial cranial CT scan obtained four weeks postoperatively showing ventricular enlargement consistent with postoperative hydrocephalus requiring CSF diversion.

Following resection of a large bifrontal parasagittal osteomeningioma (CNS WHO grade 1) and subsequent cranioplasty, a 71-year-old male developed a CSF fistula. LPS implantation resulted in complete resolution, and the patient recovered uneventfully (see Fig. 3).

### 3.5 Representative Case 3: VPS-associated infection with radiodermatitis (single-staged LPS implantation) – secondary LPS implantation

**Fig. 4:**
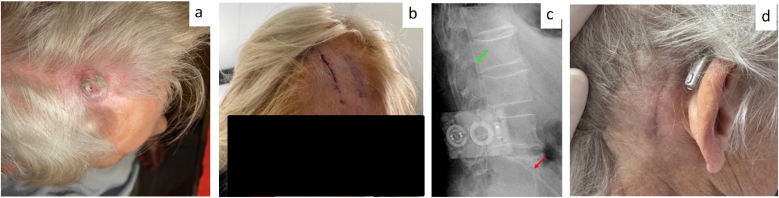
Representative Case 3: VPS-associated infection with radiodermatitis (single-staged LPS implantation) – secondary LPS implantation. **4a.** CCWC after VPS-implantation with cranial exposure of the VPS after multiple revision surgeries. **4b.** After superficial wound revision surgery with explantation of the VPS and implantation of an LPS. **4c.** Postoperative X-ray following LPS implantation. The green arrow indicates the intrathecal spinal catheter; the red arrow indicates the peritoneal catheter. **4d.** At 6-month follow-up, all wounds had healed sufficiently.

A 64-year-old female patient was diagnosed with multiple brain metastases from breast cancer. Due to leptomeningeal carcinomatosis, the patient developed communicating hydrocephalus and a VPS was implanted. Following radiation therapy, she developed a chronic superficial wound healing disorder at the frontal and retroauricular incision site, refractory to multiple revisions. After CSF infection was excluded according to our standardized diagnostic protocol, we opted for LPS implantation to protect the irradiated cranial tissue. In a single procedure, the VPS was removed, cranial wound revision was performed, and an LPS was implanted. Postoperative recovery was uneventful, with normal wound healing and radiologically confirmed correct shunt placement (see Fig. 4).

### 3.6 Representative Case 4: severe wound healing complication after decompressive craniectomy – primary LPS implantation

**Fig. 5:**
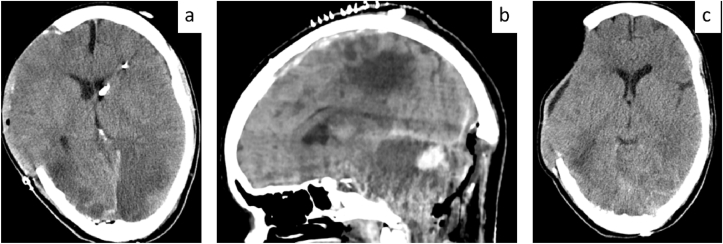
Representative Case 4: Severe wound healing complication after decompressive craniectomy – primary LPS implantation. **5a.** Axial cranial CT scan following right-sided decompressive hemicraniectomy and EVD placement in a patient with severe TBI. **5b.** Sagittal CT scan after posterior fossa decompression due to uncontrollable intracranial hypertension and transtentorial herniation. **5c.** Axial CT scan after LPS implantation demonstrating stable postsurgical conditions without signs of CSF accumulation or midline shift.

**A 31-year-old female patient was transferred to our department after sustaining a severe traumatic brain injury in a motor vehicle accident. Initial cranial CT revealed a right-sided acute subdural hematoma and extensive bilateral cerebral swelling with midline shift. Emergency right-sided decompressive hemicraniectomy and EVD placement were performed.** A few days later, the patient developed uncontrollable intracranial hypertension. Follow-up imaging demonstrated marked swelling of the posterior fossa with tonsillar herniation and transtentorial shift, necessitating posterior fossa decompression. Due to persistent hydrocephalus requiring long-term CSF diversion and anticipated CCWC resulting from the combination of supratentorial and infratentorial surgeries, a LPS was implanted. Both cranial wounds healed uneventfully, and no CSF leakage was observed during follow-up (see Fig. 5).

## 4 Discussion

Here, we present our experience with the use of an LPS system as a rescue strategy in patients with CCWC, a scenario that poses significant therapeutic challenges in neurosurgical complication management. CCWC, particularly in patients with pre-existing hydrocephalus, often involve multifactorial complications including persistent CSF fistulas, implant-associated infections, and soft tissue breakdown. The clinical course is typically characterized by repeated surgical revisions, long-term antimicrobial therapy, and extended hospital stays, all of which contribute to considerable morbidity and highly relevant socioeconomic burden.

Traditional CSF diversion via VPS is associated with relatively high revision and infection rates (Khalil et al., 2024; Conen et al., 2020; Renz et al., 2018). In their meta-analysis of VPS implantation after glioblastoma surgery, Lu et al. (2023) reported revision rates of up to 24 %, with infection representing a major cause of shunt failure and morbidity. In our cohort, three patients had a prior VPS system explanted due to wound-related complications or infection. The cranial location of VPS hardware inherently exposes the system to vulnerable tissue, thereby increasing the risk of recurrent infection and non-healing wounds.

In contrast, LPS offers distinct anatomical and functional advantages by eliminating the cranial hardware component, thereby removing the shunt system from the infected or vulnerable wound area (Palermo et al., 2025; Sun et al., 2025; Yang et al., 2019). This approach is particularly beneficial in patients with prior craniectomy, radiodermatitis, or chronic soft tissue compromise. In our series, LPS implantation resulted in complete wound resolution in all but one patient, with no recurrence of infection.

Despite its advantages, LPS implantation is associated with a distinct profile of potential complications. In our series, overdrainage represented the most frequent complication, occurring in two patients (13 %). These cases were managed conservatively by adjustment of the programmable valve, without the need for surgical revision. This observation is consistent with previous reports emphasizing the importance of individualized shunt settings in non-ventricular CSF diversion systems (Palermo et al., 2025). All LPS systems were equipped with differential pressure and gravitational units (M.Blue® or ProGAV® valves). When symptomatic or radiological signs of overdrainage – such as headache, dizziness, or small subdural hygromas – were detected, the valve pressure was increased stepwise by 2–4 units, depending on the clinical and imaging findings. Small, non-space-occupying subdural collections were treated conservatively with valve adjustment and close radiological monitoring. Sinking flap syndrome represents a rare but clinically significant complication following LP Shunt implantation. Thus gravitational valve units play a crucial role in mitigating overdrainage-related pressure gradients. Early recognition is essential, as gradual valve pressure adjustment or temporary shunt occlusion by setting the valve to its highest pressure level typically leads to rapid clinical improvement. No case of acquired Chiari malformation occurred in our cohort during the observation period. Although acquired Chiari malformation has been described as a delayed complication of chronic overdrainage in LPS-treated patients (Lovely and Peleggi, 2012; Hentati et al., 2019), it appears to be extremely rare when programmable gravitational valves are used and pressure levels are individually adjusted during follow-up.

Catheter-related complications, including distal catheter migration or disconnection, were rare. When suspected clinically (e.g., reduced drainage, local pain, or abdominal symptoms) or radiologically (via X-ray or CT), surgical revision with reconnection or repositioning of the shunt system was performed. In our cohort we experienced one single case of initial malpositioning of the peritoneal catheter. To prevent such events, all procedures were carried out under intraoperative X-ray. The peritoneal catheter was inserted under direct visualization of the peritoneum, guaranteeing correct intraperitoneal placement and tension-free tunneling. Abdominal complications (such as bowel perforation, pseudocyst formation, or peritonitis) were not observed in this series.

In summary, the most relevant complications of LPS in our cohort were related to overdrainage, which were fully manageable by non-surgical means. Through careful patient selection, initial high-pressure valve settings, using intraoperative X-ray and preoperative spinal imaging and strict adherence to our diagnostic and treatment protocols, severe complications such as acquired Chiari malformation, postoperative meningitis, or abdominal pathologies were successfully avoided.

Yadav et al. (Yadav et al.) a prospective review of 409 LPS procedures, established LPS as an effective diversion strategy for communicating hydrocephalus and catalogued the main complication spectrum (low-pressure headaches/overdrainage, CSF leak, and rare need for conversion to VPS). In their series, CSF leak at the lumbar end and occasional distal catheter issues were noted, and conversion to VPS was required in a small subset – underscoring the importance of careful patient selection and follow-up. These observations align with our practice of reserving LPS for non-obstructive cases and using programmable gravitational valves to mitigate overdrainage in CCWC patients. The broader review by Yadav et al. (2010) in 2010 summarizes indications, technique, and complication avoidance for LPS, including attention to overdrainage, catheter migration, and the rare and uncommon risk of acquired Chiari malformation. We explicitly aligned our approach with the review's emphasis on programmable valve settings and meticulous distal catheter placement, reflecting our own initial high-pressure strategy (especially in patients after decompressive hemicraniectomy) and standardized supracostal valve board positioning.

The utility of LPS in similar contexts has been corroborated by other studies (Ho et al., 2023; Kim et al., 2025; Goto et al., 2023; Sun et al., 2025; Yang et al., 2019). Yamashiro et al. (2017) reported successful use of LPS in patients with leptomeningeal metastasis–related hydrocephalus, achieving symptomatic relief without cranial intervention. Likewise, Kim et al. (2025) demonstrated in a comparative study of patients with non-small cell lung cancer and leptomeningeal metastasis that LPS implantation was associated with fewer surgical complications than VPS, though careful patient selection remains crucial.

Infection risk remains a principal concern in all forms of CSF diversion (Butenschoen et al., 2021; Aufschnaiter-Hiessboeck et al., 2024). In a prospective observational cohort study, Hussein et al. (2019) identified prior surgeries and the presence of external devices as major risk factors for postoperative meningitis in neurosurgical patients with CSF drains. By avoiding cranial and subgaleal implantation, the LPS system may mitigate these risks, as reflected in the low infection-related revision rate in our cohort.

Our study is limited primarily by its retrospective design, which inherently restricts the control over data collection, potential confounders, and standardized follow-up assessments. As a consequence, causality between LPS implantation and wound healing outcomes can only be inferred, not proven. Furthermore, as a single-center experience, our results reflect the specific surgical protocols, patient selection criteria, and interdisciplinary treatment standards established at our institution, which may not be entirely generalizable to other settings with differing expertise or patient demographics.

The small sample size (15 patients) further limits the statistical power of the analysis and the ability to perform subgroup comparisons, for example regarding underlying pathology or valve type. Nonetheless, the relative homogeneity of the cohort – defined by the shared feature of complex cranial wound conditions requiring durable CSF diversion – provides internal consistency and supports the relevance of the findings within this highly selected patient group.

We also acknowledge the possibility of selection bias, as only patients deemed suitable for LPS according to strict infection-control and anatomical criteria were included. This may have contributed to the favorable outcomes and low complication rates observed. Prospective multicenter studies with larger sample sizes and standardized evaluation protocols are needed to confirm these findings and assess the long-term durability of LPS in the management of complex cranial wound conditions.

In summary, while our results demonstrate promising outcomes for LPS as a salvage strategy in CCWC, they should be interpreted within the context of these methodological constraints.

## 5 Conclusion

LPS represents a valuable salvage option in the management of CCWC, particularly in patients with recurrent infections, CSF fistulas, or irradiated cranial tissue. By eliminating cranial hardware, this approach enables durable CSF diversion while promoting wound healing and reducing the risk of reinfection. In our cohort, LPS implantation achieved a 100 % implant survival rate and led to stable wound conditions in 14 of 15 patients. All complications were manageable either conservatively or with minor revision. These findings support LPS as a safe and effective alternative to conventional CSF diversion techniques in selected patients with complex cranial wound healing disorders.

## Author contributions

Conception and design: Vincent Prinz, Marcus Czabanka, Lina-Elisabeth Qasem

Acquisition of data: Christian Seemann, Lina-Elisabeth Qasem, Jan Oros, Sven König, Kristin Lucia, Fatma Kilinç, Tobias Finger, Paul Kendlbacher, Christoph Hirche

Analysis and interpretation of data: Christian Seemann, Lina-Elisabeth Qasem

Drafting the manuscript: Christian Seemann, Lina-Elisabeth Qasem, Vincent Prinz.

All authors reviewed the results and approved the final version of the manuscript.

## Data availability statement

The datasets used and analyzed during the current study are available from the corresponding author on reasonable request.

## Conflict of interest statement

VP and TF have received lecture honoraria from BBraun - Aesculap AG company in the past, not related to this study. The manufacturer was not involved in study design, data collection, analysis, or manuscript preparation. The authors have no personal, financial, or institutional interest in any of the drugs, materials, or devices described in this article.
